# Identification of *Phosphoglycerate Kinase 1 *(*PGK1*) as a reference gene for quantitative gene expression measurements in human blood RNA

**DOI:** 10.1186/1756-0500-4-324

**Published:** 2011-09-06

**Authors:** Virginia R Falkenberg, Toni Whistler, Janna' R Murray, Elizabeth R Unger, Mangalathu S Rajeevan

**Affiliations:** 1 Division of High-Consequence Pathogens and Pathology, Centers for Disease Control & Prevention, Atlanta, GA, 30333, USA

## Abstract

**Background:**

Blood is a convenient sample and increasingly used for quantitative gene expression measurements with a variety of diseases including chronic fatigue syndrome (CFS). Quantitative gene expression measurements require normalization of target genes to reference genes that are stable and independent from variables being tested in the experiment. Because there are no genes that are useful for all situations, reference gene selection is an essential step to any quantitative reverse transcription-PCR protocol. Many publications have described appropriate genes for a wide variety of tissues and experimental conditions, however, reference genes that may be suitable for the analysis of CFS, or human blood RNA derived from whole blood as well as isolated peripheral blood mononuclear cells (PBMCs), have not been described.

**Findings:**

Literature review and analyses of our unpublished microarray data were used to narrow down the pool of candidate reference genes to six. We assayed whole blood RNA from Tempus tubes and cell preparation tube (CPT)-collected PBMC RNA from 46 subjects, and used the geNorm and NormFinder algorithms to select the most stable reference genes. *Phosphoglycerate kinase 1 (PGK1) *was one of the optimal normalization genes for both whole blood and PBMC RNA, however, additional genes differed for the two sample types; *Ribosomal protein large, P0 (RPLP0*) for PBMC RNA and *Peptidylprolyl isomerase B *(*PPIB) *for whole blood RNA. We also show that the use of a single reference gene is sufficient for normalization when the most stable candidates are used.

**Conclusions:**

We have identified *PGK1 *as a stable reference gene for use with whole blood RNA and RNA derived from PBMC. When stable genes are selected it is possible to use a single gene for normalization rather than two or three. Optimal normalization will improve the ability of results from PBMC RNA to be compared with those from whole blood RNA and potentially allows comparison of gene expression results from blood RNA collected and processed by different methods with the intention of biomarker discovery. Results of this study should facilitate large-scale molecular epidemiologic studies using blood RNA as the target of quantitative gene expression measurements.

## Background

High-throughput and quantitative gene expression studies are carried out to identify biomarkers and determine the pathophysiology of many complex diseases. Blood is often studied as a systemic sensor of multi-system pathology, particularly in situations where anatomic lesions are lacking or inaccessible such as chronic fatigue syndrome (CFS) and neuropsychiatric diseases [1-4]. Peripheral blood is a convenient specimen for molecular epidemiologic studies and is a standard clinical sample as it is collected with minimally invasive procedures.

Blood collection and processing methods vary. Peripheral blood mononuclear cells (PBMC) may be isolated using either ficoll-hypaque purification methods or cell preparation tubes (CPT). However, isolating PBMCs requires sample processing within several hours of collection to assure RNA integrity and preservation of expression profile. Each of the isolations requires significant hands-on time and up to four hours to complete, making the equipment and personnel requirements for closely spaced time series impractical. Isolation of PBMCs may also lead to *ex-vivo *changes in their gene expression profile [5,6]. To overcome these limitations, blood collection systems that allow immediate lysis of cells and preparation of nucleic acids without prior isolation of PBMCs are now available from BD Diagnostics (Paxgene Blood Tube) and Applied Biosystems (Tempus Whole Blood Tube). In these methods yielding whole blood RNA, RNA stabilization and lysis buffer is mixed with the blood as it is drawn into the tube, minimizing changes from the *in-vivo *gene expression profile. These tubes also provide an advantage for field studies as the lysed whole blood can be stored at room temperature or at 4°C for 5-7 days, or it can be stored long term at -20°C [7].

PBMCs and whole blood RNA show differences in the cell populations that contribute. PBMC RNA is from mostly T-, B-, natural killer cells and monocytes. Whole blood RNA also includes neutrophils (about 70% of white blood cells), basophils, eosinophils, granulocytes and vast numbers of red blood cells, some of which are immature and retain messenger RNA [8].

A critical need in quantitative gene expression studies is the identification of reference genes (stably expressed genes) to normalize the expression of target genes in a particular sample [9-11]. While there are studies reporting the identification of reference genes for a variety of specimens, this information is lacking for blood in the context of different collection and processing methods. Studies of CFS with PBMCs used reference genes including *Phosphoglycerate kinase 1 *(*PGK1*) [12], *Peptidylprolyl isomerase B *(*PPIB*) [13], and *Glyceraldehyde phosphate dehydrogenase *(*GAPDH*) [14], while CFS studies using whole blood RNA isolated from Paxgene tubes have used *Transcription factor II B *(*GTF2B*) [15], *GAPDH *[16-18], and 18S rRNA [19]. Although many of these studies compared multiple candidate genes, only one study [12] reports using currently recognized algorithms like geNorm [10] or NormFinder [11] to quantitatively assess whether these genes are the most stably expressed. Publications from other diseases that have used these algorithms have identified *Ribosomal protein large, P0 *(*RPLP0*) as a stable reference gene for ficoll-hypaque isolated PBMC [20]; while *RPLP0 *[20], *Tyrosine 3 monooxygenase/Tryptophan 5 monooxygenase activating protein, zeta polypeptide *(*YWHAZ*) [10,21] and *PPIB *[22,23] have been validated for use with whole blood RNA isolated either by lysis of red blood cells or by using the Paxgene system. Although these studies have investigated PBMC or whole blood RNA separately, a direct comparison of results from PBMC RNA and whole blood RNA where both samples were drawn from the same subjects at the same time point has not been examined. Without this direct comparison it is difficult to evaluate if there are common reference genes suitable for both PBMC and whole blood RNA. We examined reference gene selection for quantitative reverse transcription PCR (qRT-PCR) using Tempus whole blood RNA and CPT isolated PBMC RNA from the same donors.

## Results

**Figure 1:**
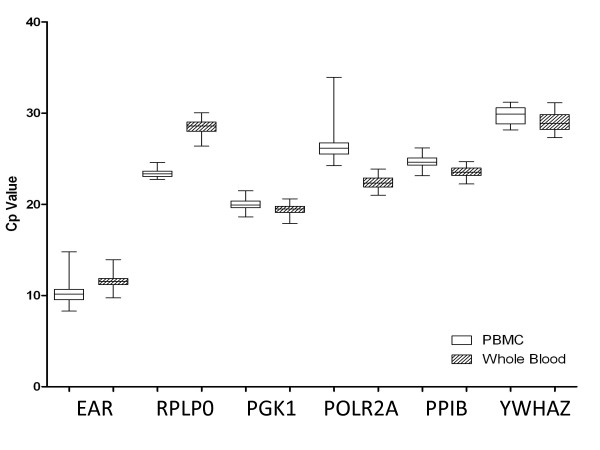
**Crossing point (Cp) values for each candidate gene in Whole Blood and PBMC RNA**. Boxes show the 25^th ^and 75^th ^percentile, with lines representing the median. The whiskers show the minimum and maximum Cp value for each gene.

The expression of candidate reference genes were measured for 46 subjects, (21 CFS subjects and 25 non-fatigued, NF, controls) using qRT-PCR. Primers and reaction efficiencies for each gene are listed in Table 1. The average crossing point (Cp) values, 25^th ^and 75^th ^percentile, and minimum and maximum values are shown in Figure 1, providing an indication of the overall stability and relative quantity for each gene. On average, Expressed Alu Repeats (EAR) was the most highly expressed and *YWHAZ *had the lowest expression in both PBMC and whole blood RNA. In terms of the standard deviations of the Cp values, genes *RPLP0*, *PGK1 *and *PPIB *appear to be the most stably expressed in PBMCs and *PGK1*, *PPIB*, and *DNA-directed RNA polymerase II polypeptide A *(220 kDa), (*POLR2A*) for whole blood RNA.

**Table 1:** Gene specific-primers and key PCR conditions.

Gene Symbol(GenBank #) Name	Primer (5'-3')*	Anneal-ing Temp (°C)	Ampli-con Size (bp)	PCR Efficiency
*YWHAZ *(NM_003406) Tyrosine 3-monooxygenase/tryptophan 5-monooxygenase activation protein, zeta polypeptide	FW: TGATCCCCAATGCTTC RV: TGTTGTGACTGATCGAC	60	127	1.81

*PPIB *(NM_000942) Peptidylprolyl isomerase B	FW: CAGCAAATTCCATCGTG RV: CCGTAGTGCTTCAGTTT	60	132	1.97

*RPLP0 *(NM_015741) Ribosomal protein, large, P0	FW: CCTTCTCCTTTGGGC RV: CCGTAGTGCTTCAGTTT	60	133	1.83

*PGK1 *(NM_000291) Phosphoglycerate kinase 1	FW: CAAGAAGTATGCTGAGGCTGTCA RV: CAAATACCCCCACAGGACCAT	58	68	1.93

*POLR2A *(NM_000937) Polymerase (RNA) II (DNA directed) polypeptide A, 220 kDa	FW: ATCTCTCCTGCCATGACACC RV: AGACCAGGCAGGGGAGTAAC	62	162	1.94

EAR Expressed Alu Repeats	FW: GAGGCTGAGGCAGGAGAATCG RV: GTCGCCCAGGCTGGAGTG	62	87	1.91

*CAB *(X56062) *Arabidopsis thaliana *chlorophyll A-B binding protein	FW: CTCAGGAATGGGCAGCACTACC RV: CAGAATCCTACAAACGCCAACAGC	60	273	1.96

*FW, forward primer; RV, reverse primer

Results of geNorm and NormFinder analyses for reference genes in PBMC and whole blood RNA are shown in Table 2, with the least stable genes at the top and the most stable at the bottom. The most variable genes as determined by the geNorm and NormFinder algorithms in PBMC RNA were EAR and *POLR2A *while *YWHAZ *and *RPLP0 *showed the most variable expression in whole blood RNA. *PGK1 *was identified as one of the two most stable reference genes for both PBMC and whole blood RNAs, whereas the second gene differed; *RPLP0 *for PBMC and *PPIB *for whole blood. The NormFinder algorithm takes into account the classification of subjects as CFS and NF but the geNorm algorithm does not; however both algorithms identified the same two genes indicating these genes are the most stable independent from disease status.

**Table 2:** Reference genes listed by increasing stability in Tempus and PBMC RNA samples.

PBMC RNA		Whole blood RNA	
**geNorm**	**NormFinder**	**geNorm**	**NormFinder**

*POLR2A*	*POLR2A*	*YWHAZ*	*YWHAZ*

EAR	EAR	*RPLP0*	*RPLP0*

*YWHAZ*	*PPIB*	*POLR2A*	EAR

*PPIB*	*YWHAZ*	EAR	*POLR2A*

*RPLP0*-PGK1*	*PGK1*	*PPIB*-PGK1*	*PGK1*

	*RPLP0*		*PPIB*

*With the geNorm method it is not possible to distinguish between the two most stable genes so these are listed together on one line.

**Figure 2:**
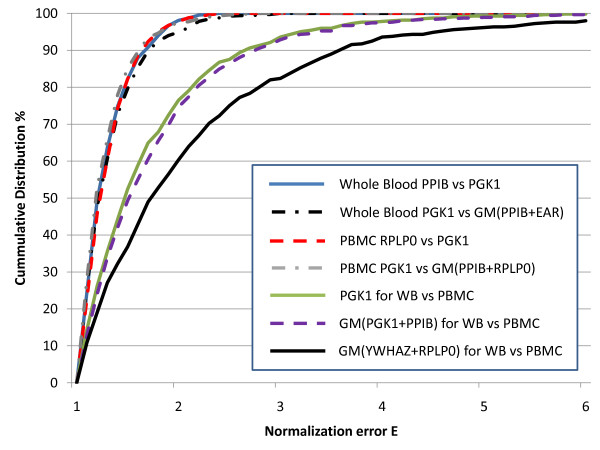
**Calculated Normalization Error (E) for single and multiple reference genes**. Normalization error values (E) were calculated as the ratio of the ratio of two reference genes in two different samples as described in Vandesompele et. al. [10] with modifications (see materials and methods) and are shown as cumulative percent distribution for each sample type. The graph shows independent measurements in Whole Blood and PBMC: Whole Blood, *PPIB *vs. *PGK1*-blue solid line; PBMC, *RPLP0 *vs. *PGK1*-red dashed line. These overlap with the comparisons to the calculated geometric mean (GM) of the two most stable reference genes: Whole Blood, *PGK1 *vs. GM (*PPIB *and EAR)-black dashed line or PBMC, *PGK1 *vs. GM (*RPLP0 *and *PPIB*)-grey dashed line. For these within-tissue comparisons, the fold error for 90% of the samples is 1.7 or less. In the cross-tissues analysis where PBMC was compared to Whole Blood, the fold error for 90% of the samples is much higher at 2.7. The fold-difference between PBMC vs. Whole Blood normalized by *PGK1 *is shown as a solid green line, the geometric mean of *PPIB *and *PGK1 *is a dashed purple line, and the geometric mean of *YWHAZ *and *RPLP0 *is shown as a solid black line.

To determine whether a stably expressed single gene is sufficient for normalization of expression studies using blood RNA we used the measure of single control normalization error (E), first described by Vandesompele et al. [10] with the geNorm algorithm. E was calculated as the ratio of the ratio of two reference genes for every combination of two samples and shown as cumulative percent distribution (Figure 2). The value of E can be interpreted as the fold-error in expression (ideally 1.0) between pairs of samples, depending on the reference gene used. We followed the standard E calculation method to illustrate the normalization error contributed by the single reference gene (see data analysis section). Based on this, for the most stable genes in PBMC RNA, *RPLP0 *vs *PGK1*, E was low with 75% of samples being less than 1.4-fold different and 90% of samples under 1.6-fold (Figure 2, red dashed line). A similar magnitude of error was observed for stable genes *PPIB *vs *PGK1 *in whole blood RNA (Figure 2, blue solid line). To assess the impact of using multiple reference genes, we modified the standard E algorithm where the expression of one gene was replaced by the geometric mean of multiple reference genes (i.e., comparing *PGK1 *to the geometric mean of *PPIB *and *RPLP0*). If the use of multiple reference genes does not change the E value then there is no advantage to using multiple genes instead of a single gene. It is important to avoid comparing a single gene (i.e., *PGK1*) to any combination of genes that includes itself (i.e., *PGK1 *+ *RPLP0*). Accordingly, we compared *PGK1 *to the geometric mean of *PPIB *and *RPLP0 *for PBMC (Figure 2, grey dashed line) or *PGK1 *to the geometric mean of *PPIB *and EAR for whole blood (Figure 2, black dashed line). These distributions essentially overlap with those of the single gene comparisons with PBMCs or whole blood RNA, indicating little improvement in minimizing error by including additional reference genes.

To further evaluate the use of single versus multiple reference genes, E was calculated to compare the expression of *PGK1 *in whole blood vs. PBMC of each subject. Because this comparison was between the two tissue types, the gene stability from a combined dataset including both whole blood and PBMC data was re-evaluated using geNorm. The most stable genes across tissues were *PPIB *and *PGK1 *while the least stable genes were *RPLP0 *and *YWHAZ*. The distributions for these comparisons shifted to the right where 70% of samples had < two-fold error and 90% of samples had < three-fold error. The use of a single gene, *PGK1*, (Figure 2, green solid line) results in lower values of E than the geometric mean of the most stable (*PGK1 *and *PPIB*) (Figure 2, purple dashed line) and unstable (*YWHAZ *and *RPLP0*) (Figure 2, black solid line) gene pairs. The trend for higher E values when additional reference genes are included also supports the use of a single reference gene for normalization.

## Discussion

From a panel of candidate genes selected using information from our own microarray studies and the published literature this study identified *PGK1 *as one of the most stable reference genes for both PBMC and whole blood RNAs by geNorm and NormFinder, two widely used algorithms based on different principles for reference gene selection [10,11]. Additional support for the use of *PGK1 *as a stably expressed reference gene is provided by the minimal standard deviation in the Cp values (Figure 1), and the essentially overlapping E-value distributions with the single and multiple gene comparisons (Figure 2).

The geNorm algorithm is based on pair-wise comparison of genes in a set of samples and selects genes with the most stable expression ratio. One drawback to this method is that two genes may have stable expression ratios because they are coordinately regulated. Since we selected candidate genes from different functional classes, contribution of coordinate regulation in the stability index is unlikely. The NormFinder algorithm provides a direct measure of variability in expression, within and between groups and confirms that there is not any inter-group variability (i.e. between cases and controls) in the expression of the candidate gene. Considering some fundamental differences between these two algorithms, it is interesting that they identified the same pairs of stable genes for both PBMC RNA (*RPLP0 *and *PGK1*) and whole blood RNA (*PPIB *and *PGK1*).

The widely recognized geNorm algorithm argues for the use of multiple reference genes for normalization of qRT-PCR through the measure of single control normalization error, E[10]. This earlier report showed considerable variation in single gene expression in a variety of tissues (3 and 6.4 fold at the 75^th ^and 90^th ^percentile respectively). In our study with PBMC and whole blood RNA, the calculated E for our two most stable single gene measures have much lower fold-differences where our plots are closer to the systematic error plotted in Vandesompele et al., with a 75^th ^percentile of 1.4 and 90^th ^percentile of 1.7. This shows quantitatively that the basis for requiring multiple genes for normalization is dependent on the stability of the candidate genes and in the case where stable genes are selected, it is not necessary to use more than one gene for data normalization. A limitation of this analysis is the inability to compare *PGK1* to a normalization factor that includes *PGK1* and the second most stable gene within a single tissue type. However, it is not a limitation when using the E algorithm to evaluate whole blood and PBMC samples from the same subject. The use of a single gene, *PGK1 *and the two most stable genes (*PGK1 *and *PPIB*) had similar distributions in this cross-tissues analysis further supporting the use of a single gene for normalization. The higher values of E for these comparisons across two different tissue types are expected and indicate that < 3-fold differences are not likely to be meaningful when doing this kind of cross-tissue comparison.

The most variable genes identified in Tempus whole blood RNA include *YWHAZ *and *RPLP0*; contradicting previous whole blood RNA studies using the Paxgene system [10,20,21]. This discrepancy can be explained by the use of a different pool of candidate genes and our selection of candidates previously identified as stably expressed. The reference genes EAR and *POLR2A *were the most variable genes in PBMC RNA in this study. *POLR2A *was selected based on preliminary analysis of microarrays done using Tempus whole blood RNA. We included EAR as a potential reference standard based on a recent report [24] that advocated the use of these widely expressed 300 bp repeat sequences as a global normalization method based on analysis of whole blood RNA from Paxgene tubes. The high variability of EAR and *POLR2A *in PBMC RNA may reflect differences in cell populations, and in whole blood the high percentage of a single cell type (neutrophils) may contribute to the reduced variability of these genes.

## Conclusion

We have identified stable reference genes for use with whole blood RNA and RNA derived from PBMC. When stable genes are selected it is possible to use a single gene for normalization rather than two or three. The identification of *PGK1 *as a suitable reference gene for both PBMC and whole blood RNA, despite differences in cell composition, shows there is clearly a relationship between these two samples. Optimal normalization will improve the ability of results from PBMC RNA to be compared with those from whole blood RNA and potentially allows comparison of gene expression results from blood RNA collected and processed by different methods with the intention of biomarker discovery. However, normalization will never fully compensate for the differences in sample composition. Investigators must recognize that while both samples originate from peripheral blood, they are actually different "tissues" because of cell fractionation that occurs during processing. Results of this study should facilitate large-scale molecular epidemiologic studies using blood RNA as the target of quantitative gene expression measurements.

## Materials and methods

### Subjects

The samples used in this analysis were obtained from a population based study of CFS in Georgia, USA that adhered to human experimental guidelines of the U.S. Department of Health and Human Services and the Helsinki Declaration. The CDC Human Subjects committee approved the study protocol, and all subjects gave informed consent. Our analysis included samples from 46 subjects (21 CFS and 25 NF), a subset of total enrollment. CFS cases were identified based on the 1994 international research definition of CFS as operationalized by standardized questionnaires including the Multidimensional Fatigue Inventory, the SF-36^® ^Health Survey, and the CDC symptom inventory [25].

### Gene Selection

Candidate reference genes were selected by three separate methods. Four genes were selected based on careful review of the literature focused on previous analyses of genes suitable for gene expression studies using PBMC: *YWHAZ*, *HUPO*, *PPIB*, and *PGK1 *[12,13,20,23]. Because we could not find published studies examining what genes might be suitable for analysis of Tempus RNA, we used preliminary data from microarray analysis of these samples to select *POLR2A*. We used Tempus RNA for global gene expression analysis using the Human Exon Array (Affymetrix, CA). In this population-based case-control study, each subject has array results from blood collected into Tempus tubes at several time points. Results from preliminary microarray analysis were averaged and the coefficient of variation (CV) calculated for subjects grouped by either disease status or by time point. In order to select a gene that is not differentially expressed over the time course of the experiment or by disease status; we selected the least variable gene that had approximately equal CV in both. Based on this analysis *POLR2A *was selected as a candidate endogenous control for Tempus RNA samples. *POLR2A *was the 18^th ^least variable gene out of 17,629 genes when based on the time course alone, and the 590^th ^least variable gene when sorted based on disease status alone. We included EAR based on a recent report advocating the utility of EAR as a potential endogenous control [24] for human blood because these repetitive 300 bp Alu sequences are scattered throughout the human genome with apparently no tissue-specificity in their expression. Table 1 shows the panel of candidate reference genes, exogenous spike-in transcript and their PCR primers. We used published primer sequences for EAR, *PPIB*, and *PGK1 *whereas we designed primers for *POLR2A*, *RPLP0*, and *YWHAZ *genes using Primer 3 v.0.4.0 [26]. Except for EAR, all gene-specific primers are intron-spanning.

### Blood collection and RNA extraction

Blood was drawn into Tempus blood tubes (ABI-Life Technologies, CA) (3 mL) or CPT (BD Biosciences, CA) (8 mL), and transported to the laboratory under controlled temperature conditions. CPTs were processed to isolate PBMCs according to the manufacturer's instructions within 1.5 - 4 hours of the blood draw. PBMCs were frozen in RPMI 1640 media supplemented with 10% (v/v) fetal calf serum, 100 IU/mL penicillin, 100 μg/mL streptomycin, 2 mM L-glutamine and 10% (v/v) DMSO, and stored in liquid nitrogen until use (approximately 9 months). RNA was isolated from PBMC, 5 × 10^6 ^cells per sample, using Trizol following the manufacturer's protocol (Sigma, MO). Tempus tubes were frozen at -20°C until extraction (< one month). RNA was extracted from Tempus tube blood using the 5 PRIME Perfect Pure RNA Cultured Cell Kit (Fisher, PA). For all samples, RNA quality and quantity were assessed using Agilent 2100 Bioanalyzer RNA Nano Chips (Agilent Technologies, CA) and a Nanodrop 1000 spectrophotometer (Thermo Scientific, DE). Nanodrop concentrations were used to set up reactions.

### Reverse Transcription (RT)

RNA (500 ng) was DNase I treated in a 10 μl volume using the MessageClean^® ^Kit (GenHunter, TN) and then reverse transcribed in the same tubes using 20 μl reactions with Superscript™ III (Invitrogen, CA) and a combination of Oligo(dT) and random hexanucleotide primers (2.5 μM each) in the presence of 25 pg of a plant gene spike-in *chlorophyll a/b binding protein *(*CAB*) mRNA. (Stratagene, CA). Two microliters of the RT reaction was removed prior to the addition of RT enzyme to serve as no-RT control for detecting DNA contamination. HeLa cell cDNA was synthesized to serve as template for a standard curve in the LightCycler reactions.

### PCR

PCR (20 μl) was performed using the LightCycler 480 with the SybrGreen 480 Master Mix (Roche Applied Sciences, IN) and contained 2 μl of 1:20 dilution of cDNA and 0.5 μM of each primer. Thermal cycling conditions were as follows: 1 cycle of 94°C for 5 minutes, 50 cycles of 94°C 15 seconds, annealing temperature (specific for each transcript in Table 1) for 15 seconds, and 72°C 15 seconds. Cp values were determined using the Roche LightCycler software (v1.5.0 SP1) by the second-derivative maximum method for each of the amplifications and compared to a standard curve created from six 5-fold dilutions of HeLa cell cDNA, starting at 1.25 ng/μl. Efficiency of each reaction determined from the standard curve is shown in Table 1. The external control plant gene *CAB *had an average Cp value of 19.38 ± 0.56 (CV, 2.9%). The variability of *CAB *measurements was within 1.5 fold for > 90% of samples.

### Data analysis

The standard curve normalized values for expression of each of the 6 candidate genes were used in two algorithms (geNorm and NormFinder) to determine the most stable genes for both PBMC and whole blood RNA. geNorm version 3.5, is a VBA applet that works with Microsoft Excel [10]. Briefly, geNorm calculates a gene stability measure (M) for each gene where M value increases with decreasing stability of gene expression. The gene with the least stability is removed from the analysis and M values are recalculated until the two most stable genes remain. A normalization factor is calculated based on the geometric mean of the selected reference genes. The NormFinder algorithm v 0.953 [11] uses a statistical model to estimate the overall expression variation for each candidate gene. This generates a stability value that is related to the systematic error that would be introduced when using each candidate for normalization.

The single control normalization error or E was originally described by Vandesompele et al. [10]. We have used this algorithm to compare the use of a single reference gene (*PGK1*) to the use of a normalization factor consisting of the geometric mean of two reference genes. The modified algorithm we used is as follows: for any given ***m ***subjects (samples), gene expression levels from real-time PCR of selected reference genes are measured (***a***). For every combination of two subjects, ***p ***and ***q ***and a combination of reference genes ***j ***and ***k ***the measurement error for ***j ***vs ***k ***or the E value is calculated and represents the fold-expression difference between samples ***p ***and ***q ***when normalized to ***j ***vs ***k***. The equation is R_jkpq _= a_qj_/a_qk _*a_pk_/a_pj _(if ***R ***< 1 then E = ***R***^-1^, else E = ***R***). For example when comparing two distinct reference genes such as *PGK1 *and *PPIB*, in samples 1 and 2, the resulting equation is ***R ***= (*PGK1 *in sample 1/*PPIB *in sample 1) * (*PPIB *in sample 2/*PGK1 *in sample 2) (if ***R ***is less than one take the reciprocal ***R***^-1 ^to equal E otherwise ***R ***= E). This equation PGK1/PPIB * *PPIB/PGK1 *should equal 1 if the measurements of *PPIB *and *PGK1 *are constant in both samples. The same equation is performed for all possible comparisons of samples and the cumulative distribution of the E value or fold error are plotted for Figure 2. We further modified this equation so that instead of assessing two single reference genes, we could look at the E value when a normalizing factor (such as the geometric mean of multiple reference genes) is used. This was accomplished by replacing either ***j ***or ***k ***with the geometric mean of multiple genes as calculated for that sample. The new equation then essentially measures *PGK1*/(geometric mean of *PPIB *and *RPLP0*) * (geometric mean of *PPIB *and *RPLP0)*/*PGK1 *and this equation would also be equal to 1 if expression of both the genes was constant. Clearly as this equation is based on the ratio of ***j ***and ***k ***then it is not appropriate to include the same gene as a part of both ***j ***and ***k***. (i.e., it is not meaningful to compare *PGK1*/(geometric mean of *PGK1 *and *PPIB*)*(geometric mean of *PGK1 and PPIB*)/*PGK1 *as it will be biased because *PGK1 *is included in the numerator and denominator). Therefore, to select the most stable two genes to create a normalization factor for comparison we resubmitted the standard curve normalized data to geNorm without *PGK1 *and selected the two most stable remaining genes, *PPIB *and *RPLP0 *for PBMC and *PPIB *and EAR for whole blood.

For cross-tissues comparison, E was calculated by substituting the variables ***j ***and ***k ***for PBMC or whole blood values of expression for a reference gene or the geometric mean of multiple reference genes for each subject. The resulting equation is ***R ***= (*PGK1 *in subject 1 PBMC/*PGK1 *in subject 1 whole blood) * (*PGK1 *in subject 2 whole blood/*PGK1 *in subject 2 PBMC) (if ***R ***is less than one take the reciprocal ***R***^-1 ^to equal E otherwise ***R ***= E). For determining the impact of multiple reference genes in the cross tissue comparison, we combined both PBMC and whole blood expression values into a single file and then used geNorm to identify the most stable (*PGK1 *and *PPIB*) and least stable (*RPLP0*, *YWHAZ*) genes.

## Competing interests

The authors declare that they have no competing interests.

## Authors' contributions

VRF carried out the gene selection, qRT-PCR and analysis, and drafted the manuscript. JM carried out the RNA preparation and quality analysis. TW contributed microarray data used in gene selection. VRF, MSR, TW and ERU participated in the design and interpretation of the study. ERU, MSR and VRF conceived and coordinated the study. All authors contributed to manuscript writing, and read and approved the final manuscript.
